# Research progress in intracranial artery dissections over past 25 years: a review and bibliometric analysis

**DOI:** 10.3389/fneur.2025.1623956

**Published:** 2025-06-04

**Authors:** Zixuan Zhou, Bingyang Qin, Yang Chen, Ren Li, Biao Yang, Ziao Li, Yongqiang Wu, Xiaogang Wang, Xiaolong Guo, Wenju Zhang, Yuanli Zhao, Geng Guo

**Affiliations:** ^1^Department of Emergency, First Hospital of Shanxi Medical University, Taiyuan, Shanxi, China; ^2^Cerebrovascular Disease Center, First Hospital of Shanxi Medical University, Taiyuan, Shanxi, China; ^3^Center for Cerebrovascular Diseases Research, Shanxi Medical University, Taiyuan, Shanxi, China; ^4^Department of Neurosurgery, First Hospital of Shanxi Medical University, Taiyuan, Shanxi, China; ^5^School of Public Health, Shanxi Medical University, Taiyuan, Shanxi, China; ^6^Department of Neurosurgery, Peking Union Medical College Hospital, Chinese Academy of Medical Sciences and Peking Union Medical College, Beijing, China; ^7^Shanxi Provincial Clinical Research Center for Interventional Medicine, Taiyuan, Shanxi, China; ^8^MOE Key Laboratory of Coal Environmental Pathogenicity and Prevention, Taiyuan, Shanxi, China

**Keywords:** intracranial arterial dissection, pathophysiology, hotspots, clinical diagnosis and treatments, bibliometric analysis

## Abstract

**Background:**

A bibliometric and visual analysis of articles related to intracranial artery dissection (IAD) was performed to reveal the academic developments in this research field, to better assist researchers in reviewing previous research results, exploring current scientific research hotspots and cutting-edge trends, and obtaining a global perspective on IAD research.

**Methods:**

Articles on IAD published from January 1, 2000 to March 15, 2025 were searched, screened, and downloaded using the Web of Science Core Collection (WoSCC). All literature included in the study was analyzed using VOSviewer, CiteSpace and Microsoft Excel.

**Results:**

A total of 1,130 articles were included in the bibliometric analysis from January 2000 to March 2025, with a general upward trend in the number of articles published each year, peaking in 2019 for annual publications. Collaboration network analysis showed that the United States, Japan, and Germany are the more influential countries in the field, with high numbers of published articles, citations, and collaborations with other countries. The journal with the most publications was World Neurosurgery. Lawton MT was the most active author with a cumulative total of 13 articles, while Biller J received the highest number of citations with a cumulative total of 1,033. Keyword analysis of the literature showed that “recanalization “reached the highest intensity of outbreaks, while “thrombectomy “,” outcome “, “risk “, and “diagnosis “showed an increase in citations in 2025, indicating that these subjects continue to be of significant interest.

**Conclusion:**

This study explores the evolving research trends and challenges in the area of IAD, presenting a thorough examination of both its historical and current research contexts. It offers valuable direction for future scholarly pursuits. By engaging in comprehensive research and examining various perspectives within the IAD domain, new insights can be uncovered, ultimately facilitating precise diagnosis and effective treatment of the condition.

## 1 Introduction

Intracranial artery dissection (IAD) is a cerebrovascular lesion that is characterized by the tearing of the intima-media of the arterial wall and the subsequent infiltration of blood into the vessel wall, resulting in the formation of a pseudo-lumen (1). IAD can lead to the formation of pseudoaneurysms, which account a percentage of intracranial aneurysms ranging from 2 to 10% and occurs in the intracranial segment of the internal carotid artery, the basilar artery, and the vertebral artery, among other important vessels (2). The majority of IAD is spontaneous, with headache as the main symptom in 70–90% of cases, often described as “thunderclap headache.” In addition, IAD can lead to highly disabling ischemic stroke or subarachnoid hemorrhage with a mortality rate of up to 50% (3, 4). Current major treatment options for IAD include antiplatelet or anticoagulation therapy and endovascular intervention.

Since the year 2000, academic research on the effects of IAD has shown a growing trend and has mainly focused on case reports and exploration of pathologic mechanisms. With the advancement of imaging technology, the application of nascent imaging techniques such as high-resolution MRI and vessel wall imaging in the diagnosis of IAD has matured, which can produce good visualization against the arterial wall and significantly improve the early diagnosis rate of IAD (5). In recent years, a large number of studies have demonstrated that the application of flow diverter devices significantly improves the treatment outcome of pseudo-complex aneurysms secondary to arterial dissection, but its postoperative in-stent thrombosis and bleeding complications caused by antiplatelet regimens remain a clinical challenge (6). Although the number of relevant studies is increasing year by year, the existing literature mostly focuses on case reports and lacks bibliometric analysis to sort out research trends and knowledge evolution. Consequently, it is imperative to methodically organize the prevailing research trends in IAD.

By quantitatively analyzing the temporal or spatial distribution of the literature, author collaboration networks, keywords co-occurrence, and citation associations, bibliometrics can objectively reveal the developmental lineage of a discipline and the cutting-edge direction of research hotspots. Although its application in the field of cerebrovascular diseases is gradually increasing, such as intracranial aneurysms, ischemic stroke, etc., no study has yet conducted a comprehensive bibliometric analysis for the subcategory of IAD (7). Therefore, this study was conducted to systematically review and summarize the existing literature on IAD based on bibliometric research techniques.

## 2 Materials and methods

### 2.1 Data acquisition

**Figure 1:**
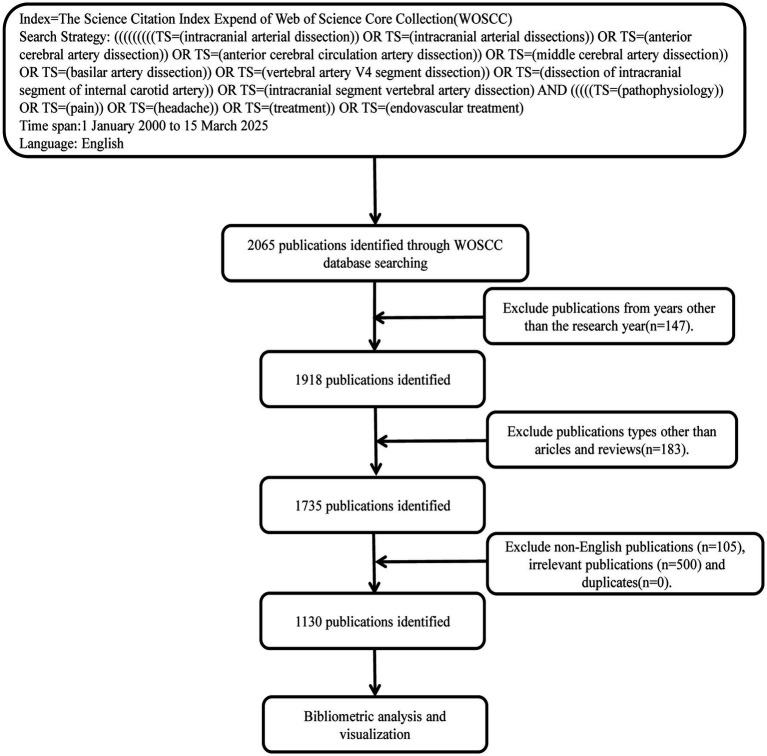
Flowchart for screening publications for this study.

This study was searched in the WoSCC on March 15, 2025, and included articles published from January 1, 2000, to March 15, 2025 on IAD. Science was searched using wildcards and the search formula is shown in Table 1. The article types of original research articles, review articles and English-language articles were selected for subsequent analysis. Articles that were irrelevant to the study topic, incomplete, retracted and duplicates were excluded. The inclusion and exclusion of controversial articles was decided after collective consideration. The literature screening process is shown by Figure 1. All literature information was exported from WoSCC in plain text file format.

**Table 1:** Search strategies.

Set	Search formula
#1	((((((((TS = (intracranial arterial dissection)) OR TS = (intracranial arterial dissections)) OR TS = (anterior cerebral artery dissection)) OR TS = (anterior cerebral circulation artery dissection)) OR TS = (middle cerebral artery dissection)) OR TS = (basilar artery dissection)) OR TS = (vertebral artery V4 segment dissection)) OR TS = (dissection of intracranial segment of internal carotid artery)) OR TS = (intracranial segment vertebral artery dissection)
#2	((((TS = (pathophysiology)) OR TS = (pain)) OR TS = (headache)) OR TS = (treatment)) OR TS = (endovascular treatment)
#3	#1 AND #2

### 2.2 Bibliometric analysis

The trend of annual and cumulative publications in this research area is statistically plotted using Microsoft Excel 2021. Citespace (version 6.4. R1) is an information visualization software based on the theory of citation analysis. It can be used to identify and display new trends and dynamics in scientific development. It can also be used to track and analyze international cutting-edge research. Furthermore, Citespace applies the “co-occurrence clustering” idea for article information processing to present different co-occurring cooperative networks and the emergence of hot keywords and cited literature. VOSviewer (version 1.6.20) is a software based on the citation and co-citation principles, which supports the construction and viewing of bibliometric knowledge maps in multiple views. The Bibliometrics Online Analysis Platform^1^ helps researchers to obtain an overall overview of all publication information in the current study, and to understand the publication status and impact of each country, author, institution, journal, and the process of topic evolution.

### 2.3 Ethics in research

The study was conducted using open databases to search and obtain publication information without the need for ethical review through an ethics committee.

## 3 Results

### 3.1 Annual analysis of trends in the number of publications and citations

**Figure 2:**
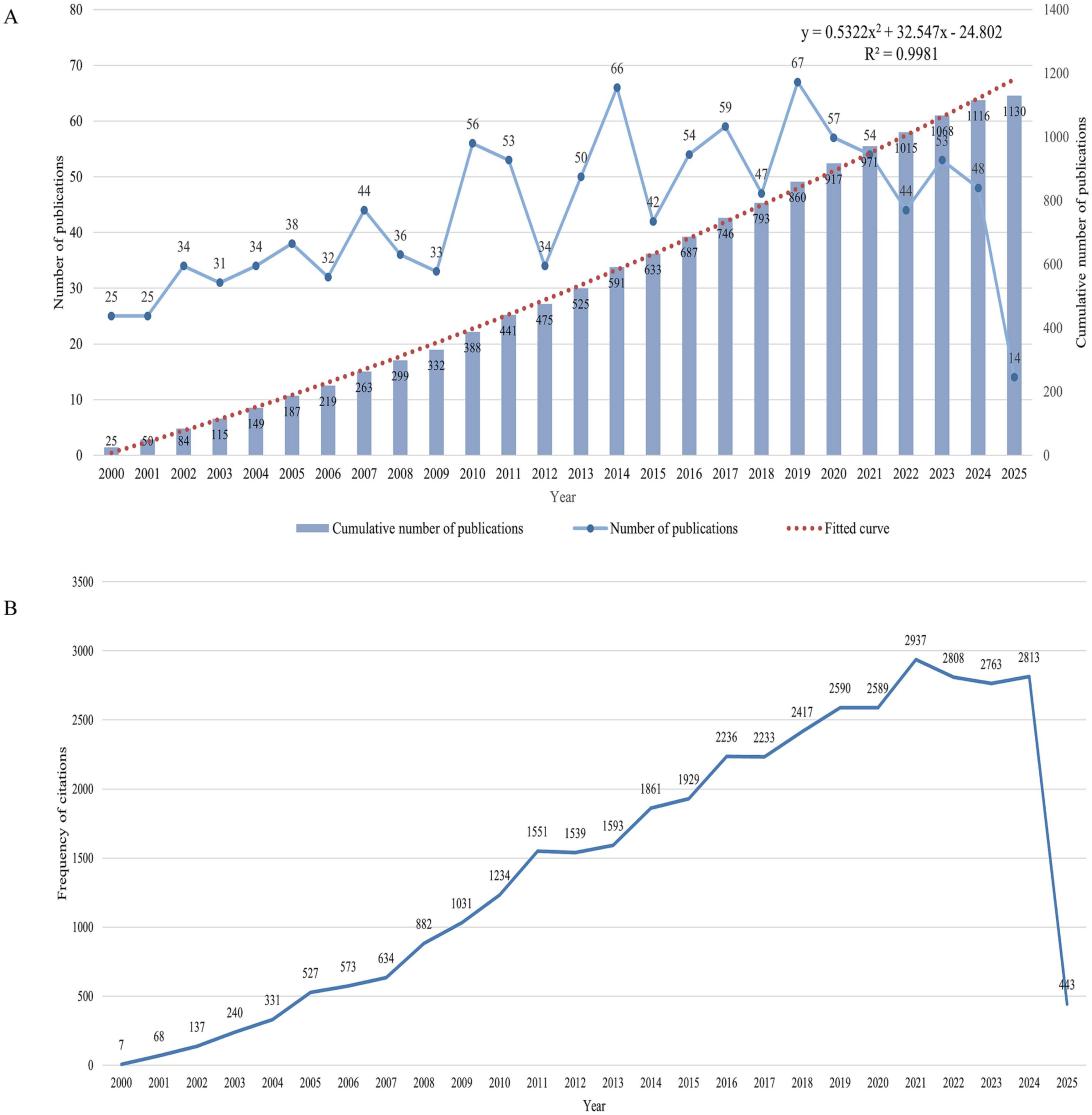
Trends in annual publication volume and citation frequency. **(A)** Trends in the number of publications, including the annual number of publications and the annual cumulative number of publications, with a polynomial fit to the annual cumulative publication trend. **(B)** Annual citation frequency curves.

A thorough search of the WoSCC database identified a total of 2,065 articles concerning IAD, spanning from January 1, 2000, to March 15, 2025. After the above series of screening,1,130 documents were finally identified for in-depth bibliometric study. Of the selected literature, 1,013 (89.7%) were original research-based articles and 103 (9.1%) were review articles. There was an overall upward trend in the number of annual publications and annual citation frequency over the established time period of the study, with an average of 43.5 annual publications and 1,460.2 annual citations. Figure 2A shows that from 2000 to 2014, the number of publications, after a brief drop in 2012 (*n* = 34), rose linearly to 66 in 2014, and in 2019 reached an annual peak of 67 posts, then decreased and fluctuated around the average number of posts. We fit a polynomial to the cumulative number of articles, and its functional expression is


$$y=0.5322x^{2}+32.547x-24.802$$

*R*^2^ = 0.9981, the larger the value of *R*^2^ indicates that the curve is fitted better, as shown by the red curve in the figure, which is a more satisfactory curve fitting effect. Since the statistics of 2025 is only up to March 15, the number of publications is only 14. Figure 2B illustrates the annual fluctuations in citation counts for publications within the IAD domain, with a very clear upward trend in the frequency of citations from 2000 to 2021, and reaching a peak of 2,937 in 2021, with a slight decrease in 2022 and 2023, and then rising again to 2,813 in 2024. The existing curves of the number of articles and the frequency of citations have certain fluctuations, so for the sake of rigor, it is difficult to predict whether the number of articles and the number of citations will continue to rise in 2025 and beyond, but we hope that experts, scholars, and clinicians in various countries will continue to explore the field of IAD in depth and achieve more research results.

### 3.2 Country/regional publications and cooperation

**Figure 3:**
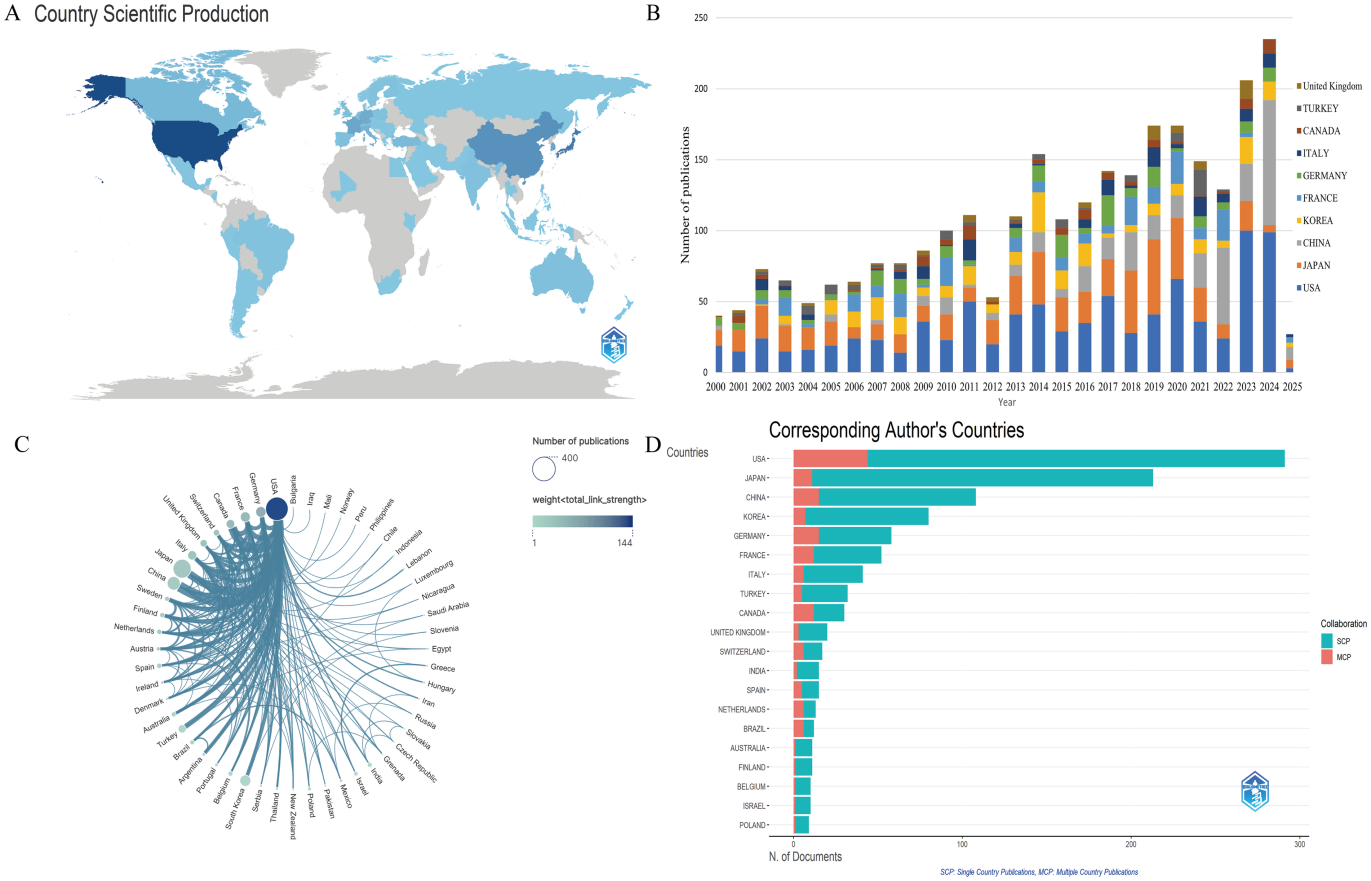
Country/regional cooperation and annual number of publications. **(A)** Map of geographical distribution of national communications. **(B)** Change in annual volume of communications for the top 10 countries in terms of volume of communications. **(C)** Chord chart for country cooperation. **(D)** Countries of corresponding author.

A total of 52 countries/regions published papers in the direction of IAD. Table 2 shows in more detail the number of publications, national collaborations, citation frequency and research impact in each country. Figure 3A visualizes the number of publications in each country combined with a map, which shows that the publications are mainly concentrated in North America, Europe and Asia. The United States tops the list with 346 publications, while Japan and China rank second and third with 222 and 116 publications, respectively. Figure 3B shows the annual number of articles by country, and combined with the data, China has the highest annual growth rate in the number of articles. In terms of total citations, the USA, surpasses Japan (*n* = 2,818) and Germany (*n* = 2,243) by an absolute margin (*n* = 9,115), and its citations are about three times as many as Japan’s and Germany’s eight times as many as Germany’s. The H-index, which is a measure of the quantity and impact of academic output, the top three are the United States (H-index = 46), Japan (H-index = 26), and the United Kingdom (H-index = 41). With high H-index, the United States and Japan indicate that their academic achievements have good influence and recognition in the field. Figure 3C shows the cooperation between countries more clearly in the form of a chord diagram, where the size of the nodes represents the number of articles published by the countries, and the thickness of the connecting line between the two countries represents the connection strength, that is, the strength of cooperation. The total connection strength listed in Table 2 is the total strength of the country’s cooperation with other countries. The strength of the connection between the United States and China is 17, the highest among all countries, followed by the United States and Canada (strength = 15), and the United States and Japan (strength = 13). Figure 3D shows the number of articles published and the collaboration of corresponding authors by country. SCP indicates the count of publications co-authored by authors sharing the same nationality, while MCP signifies those co-authored with authors from different nations. The United States leads in both SCP and MCP categories. The United States’ central role in the area of IAD is underscored by its high volume of publications and citations, significant academic influence, and extensive author collaboration and international cooperation. Cooperation between countries should be strengthened between domestic and foreign scholars to gradually improve the quality of research and publications and enhance their academic influence.

**Table 2:** Top 10 productive countries/regions in IAD ranking by the number of publications.

Rank	Countries/regions	Publications	Percentage	Citations	Total link strength	H-Index
1	USA	346	30.6%	9,115	144	46
2	Japan	222	19.6%	2,818	33	26
3	China	116	10.3%	1,136	30	15
4	South Korea	81	7.2%	1,445	9	22
5	Germany	72	6.4%	2,243	70	24
6	France	66	5.8%	2,202	55	19
7	Italy	54	4.8%	507	38	10
8	Canada	49	4.3%	1,951	54	13
9	Turkey	42	3.7%	354	11	18
10	United Kingdom	35	3.1%	1,205	38	25

### 3.3 Institutional co-occurrence and volume of publications

**Figure 4:**
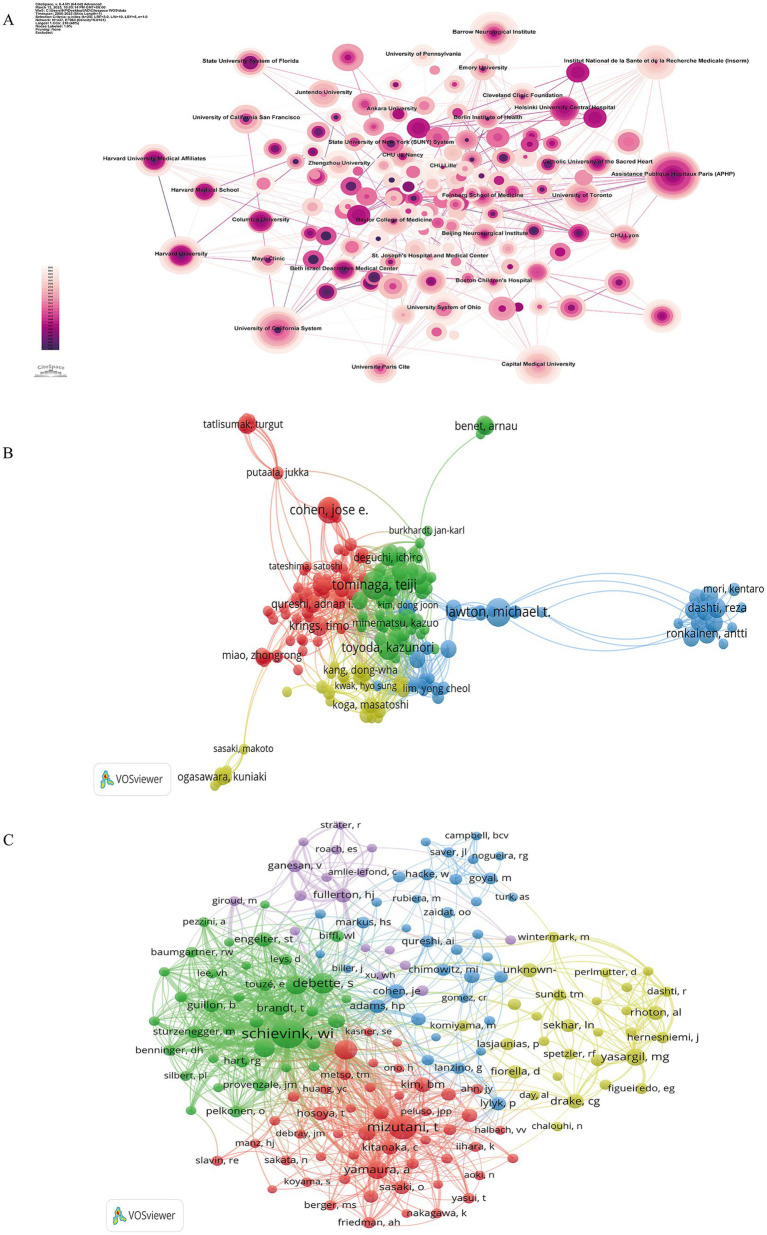
Institutions and authors co-occurrence visualization network maps. **(A)** Institutional co-occurrence visualization using Citespace, showing 963 connections from 437 institutions spanning the globe. **(B)** Collaboration network of 221 authors with more than 3 publications. **(C)** Collaborative network of 168 authors with more than 20 citations, with the node and label character size representing the number of authors’ publications, and the width of the connecting line representing the density of their collaboration.

A total of 1,499 institutions published scholarly papers in the field of IAD, and 437 had more than 3 publications. Figure 4A visualizes the collaborative co-occurrence network of research institutions, where each node represents an institution, the size of the node represents the number of publications, and the mediator centrality reflects the institution’s centrality. The larger the node, the more publications the institution has, and the larger the centrality value, the more it occupies a key position in the collaborative network. Institutions with a higher H-index tend to have greater academic prestige and impact. Table 3 lists the relevant index information of the 10 institutions with the highest number of publications. The institution with the largest number of publications is University of California System (*n* = 71), followed by Assistance Publique Hôpitaux de Paris (APHP) (*n* = 67), and Capital Medical University (*n* = 56). The institution with the highest centrality was Assistance Publique Hôpitaux de Paris (APHP) (Centrality = 0.07), with University of California System (*n* = 0.05) and University System of Ohio (*n* = 0.05) tied for second place, and Capital Medical University (*n* = 0.04) was in third place. The top three research institutions in the H-index were University of California System (H-index = 29), Assistance Publique Hôpitaux de Paris (APHP) (H-index = 28) and Université Paris Cité (H-index = 24). It is worth noting that most of the institutions with a high number of publications and occupying key academic positions are from the United States, France, and other European and American countries, and only Capital Medical University in China is among the top 10, with an outstanding publication status and academic position. The research among institutions is relatively independent, which is mainly manifested in academic cooperation among the countries they belong to.

**Table 3:** Top 10 institutions by number of publications.

Rank	Institution	Publications	Percentage	Centrality	H-Index
1	University of California System	71	6.3%	0.05	29
2	Assistance Publique Hôpitaux de Paris (APHP)	67	5.9%	0.07	28
3	Capital Medical University	56	4.9%	0.04	13
4	Université Paris Cité	45	3.9%	0.02	24
5	University of Toronto	43	3.8%	0.02	20
6	Mayo Clinic	40	3.5%	0.02	16
7	Institut National de la Sante et de la Recherche Medicale (Inserm)	36	3.2%	0.03	15
8	University System of Ohio	36	3.2%	0.05	13
9	University of California San Francisco	35	3.1%	0.01	19
10	Harvard University	33	2.9%	0.03	15

### 3.4 Author collaboration and co-citation analysis

A total of 5,826 authors contributed 1,130 articles in the field of IAD. The top 10 authors with the most published papers and the most cited frequency are shown in Table 4. Among all the authors, Lawton MT was the author with the largest number of articles published with 13 papers, which accounted for 1.2% of all. Tominaga T and Cohen JE were both in the second place with 12 papers and 1.1% of the number of articles, followed by Toyoda K (11, 0.97%) and Krings T (10, 0.88%), and all the above authors have more than 10 publications and have an H-index greater than or equal to 7. For citation frequency, the top author is Biller J (1,033 citations), followed by Kirkham FJ (927 citations), Deveber G (775 citations), Smith ER (740 citations) and Golomb MR (730 citations), with the top five authors all having a citation count greater than 700 and an H-index greater than or equal to 3. Collaboration of researchers contributes to the depth of academic research. By examining the author collaboration network, we can identify key researchers in a given field and gain insight into their collaborative efforts. Figures 4B,C visualize the collaboration network among authors. Figure 4B shows the collaboration network of 221 authors with more than 3 publications, which is more clearly shown in four clusters with four different colors. The green cluster contains the largest number of authors, and 51 authors represented by Tominaga T belong to this cluster, and the authors in this cluster studied the topics of treatment of giant carotid aneurysms and neurologic injury due to cerebral ischemia/reperfusion. The red cluster contains 50 authors and is represented by Cohen JE, whose main research interests are the use of flow diverters in cerebral aneurysms and the treatment of traumatic carotid artery dissection; the blue cluster consists of 28 authors and is represented by Lawton MT, whose research interests are the treatment of posterior circulation dissection aneurysms and post-stroke neuroinflammation; the yellow cluster is the smallest cluster and consists of 24 authors, represented by Kang DW, whose research is focused on vascular wall imaging and intracranial vascular disease imaging diagnosis. Figure 4C shows the collaborative network of 168 authors with more than 20 citations, which is shown as five clusters of red, yellow, blue, green, and purple. The size of the node and label characters reflects the number of publications by the authors, while the width of the linking line indicates the density of their collaboration.

**Table 4:** Top 10 authors with the highest number of publications and citations.

Rank	Author	Publication	H-index	Most cited authors	Citation	H-index
1	Lawton MT	13	7	Biller J	1,033	5
2	Tominaga T	12	8	Kirkham FJ	927	3
3	Cohen JE	12	7	Deveber G	775	4
4	Toyoda K	11	7	Smith ER	740	3
5	Krings T	10	9	Golomb MR	730	3
6	Kim JS	9	8	Adams R	697	1
7	Fujimura M	9	7	Daniels S	697	1
8	Inoue T	9	7	Ferriero D	697	1
9	Shimizu H	9	6	Jones BV	697	1
10	Uchino A	9	5	Roach ES	697	1

### 3.5 Analysis of journal publications and impact

**Figure 5:**
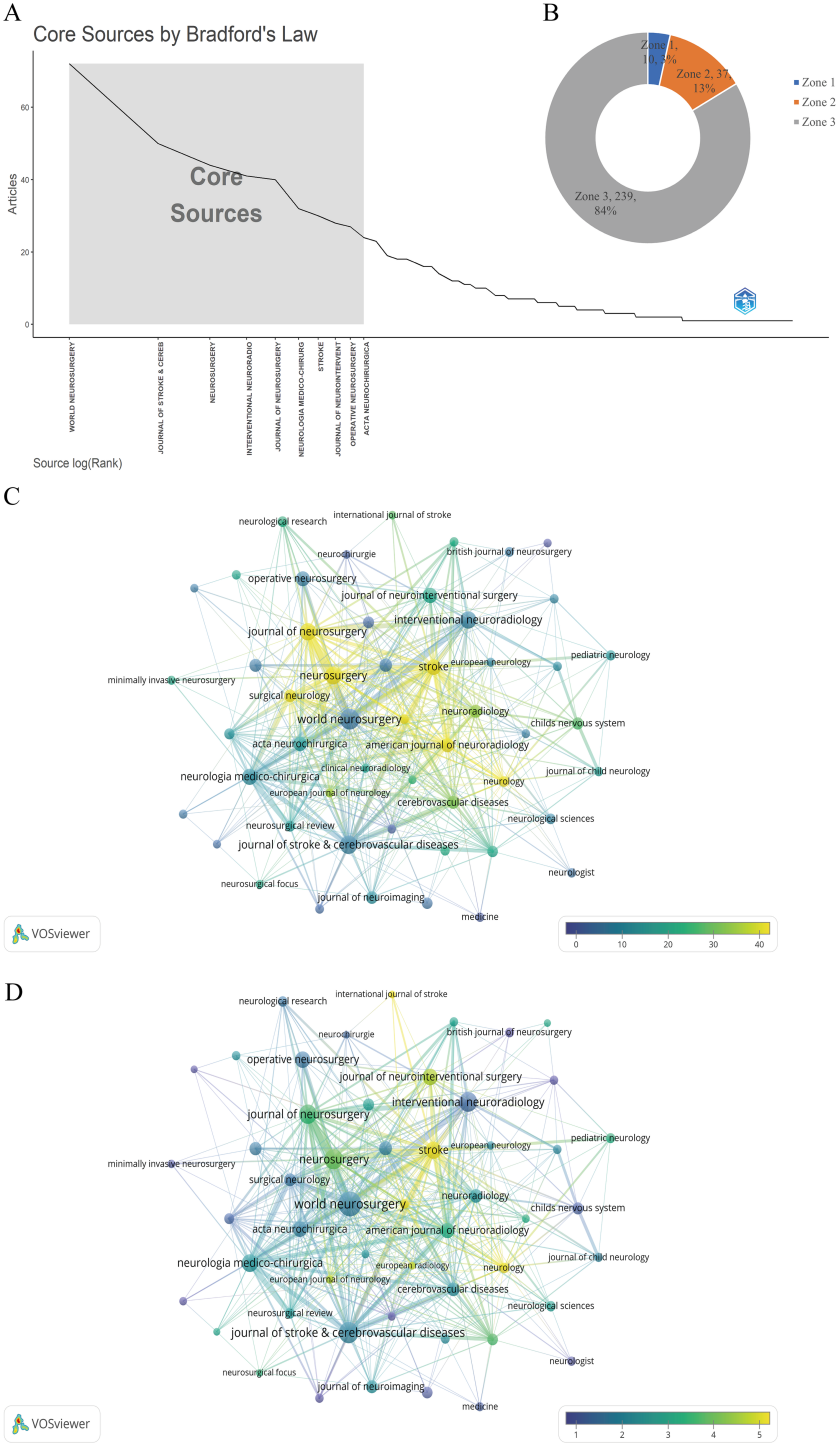
Plot of the percentage of core journals and superimposed view of journal publications with citation frequency and impact factor, respectively. **(A,B)** Number and percentage of core versus non-core journals classified according to Bradford’s law. **(C)** Superimposed network view of journal publications versus average citation frequency. **(D)** Overlay network view of journal publications versus journal impact factor.

The 1,130 articles included in the study covered a total of 286 journals. As shown in Figures 5A,B, these 286 journals were categorized into zones 1–3 according to Bradford’s law. There are 10 journals from core sources, accounting for 3.5% of the total number of journals, and the remaining two non-core zones have 37 and 239 journals, accounting for 12.9 and 83.6%, respectively. Figure 5C shows the superimposed view of the journal’s publication volume and average citation frequency, the larger the node represents the larger the publication volume of this journal, and the proximity of the node color to yellow indicates a higher average citation frequency, which indicates that the journal has a higher recognition of academic achievements in the field of IAD, and it has a more critical status. Similar to Figures 5C,D shows the superimposed view of journal publication volume and journal impact factor, the lighter the node color indicates that the higher its impact factor, the greater the academic value of the journal and the more critical its status. It can be seen that the journals with high impact factor are Stroke (IF = 7.8), Neurology (IF = 7.7), International Journal of Stroke (IF = 6.3) and so on. Table 5 lists the top 10 academic journals with the most publications. We can see that the journal with the highest number of publications in the field of IAD is World Neurosurgery with a total of 72 papers, in second place is Journal of Stroke & Cerebrovascular Diseases with 50 publications, and in third place is Neurosurgery with 44 publications. In terms of citations, the journal with the highest total citations is Stroke with a whopping 2,558 citations, followed by Neurosurgery (2,328 citations), and Journal of Neurosurgery (1,756 citations). In addition, there is another journal that deserves attention, American Journal of Neuroradiology (JCR Q2, IF = 3.1) is not included in Table 5 due to the number of articles published is only 23, but its citation frequency reaches 1,009 citations, which indicates that the academic influence of this journal cannot be ignored.

**Table 5:** Top 10 journals in terms of publications.

Rank	Journal	Publications	Total citations	Average citations	JCR	IF	H-index
1	World Neurosurgery	72	499	6.9	Q3	1.9	12
2	Journal of Stroke & Cerebrovascular Diseases	50	430	8.6	Q3	2.0	12
3	Neurosurgery	44	2,328	52.9	Q1	3.9	26
4	Interventional Neuroradiology	41	374	9.1	Q4	1.5	10
5	Journal of Neurosurgery	40	1,756	43.9	Q1	3.5	25
6	Neurologia Medico-Chirurgica	32	364	11.38	Q2	2.4	11
7	Stroke	30	2,558	85.3	Q1	7.8	22
8	Journal of Neurointerventional Surgery	28	544	19.4	Q1	4.5	14
9	Operative Neurosurgery	27	219	8.1	Q3	1.7	8
10	Acta Neurochirurgica	24	368	15.3	Q3	1.9	11

### 3.6 Analysis of research disciplines

**Figure 6:**
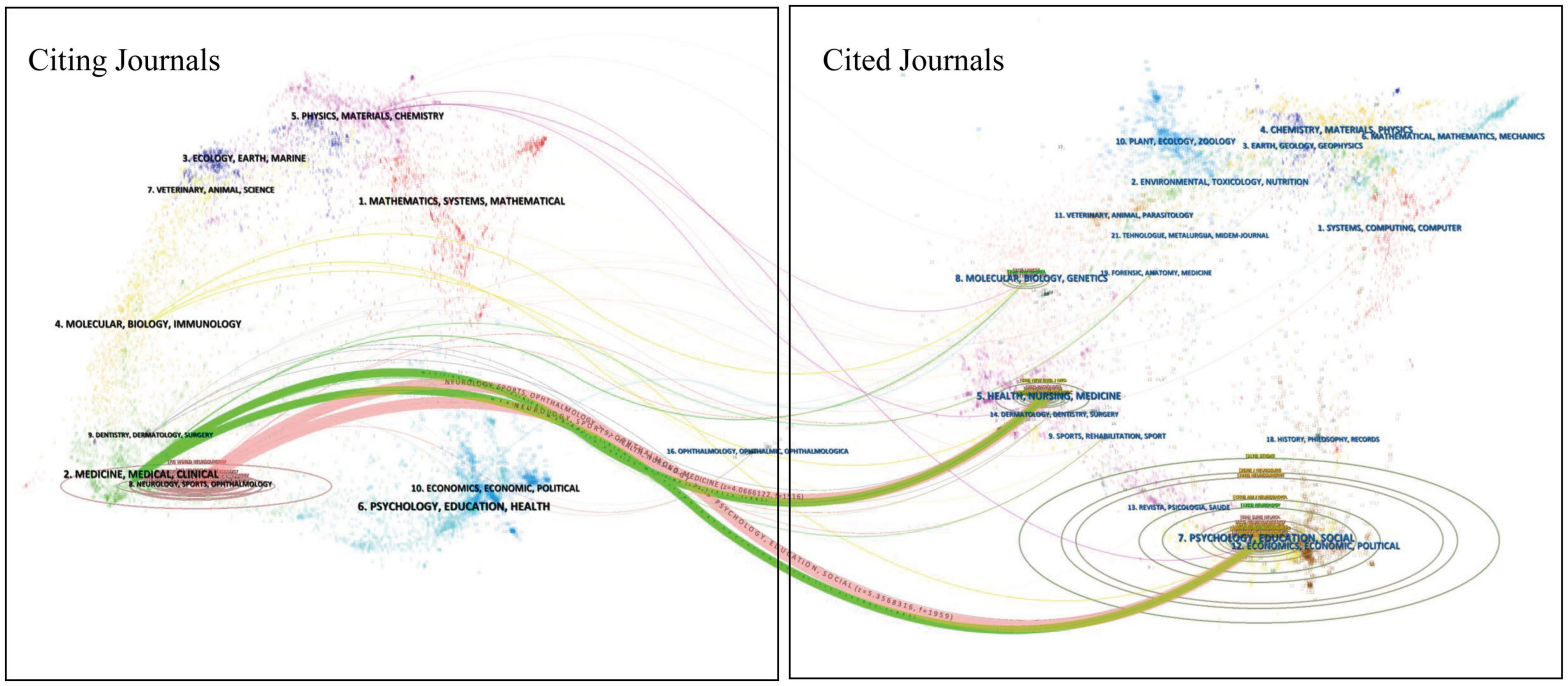
A dual-map overlay of journals on IAD.

The dual-map overlay of journals serves as a tool to depict the distribution of subjects across journals, track the evolution of citation trajectories, and identify the relocation of research centers. The study of IAD spans various disciplines and fields, as illustrated by the dual-map overlay of journals on IAD in Figure 6. On the left side, the distribution of major journals in the WOSCC database for research on IAD is shown as a collection of citing journals, depicting the present state of knowledge within the discipline of research; the more papers a journal publishes, the longer the ellipse’s longitudinal axis becomes; similarly, the greater the number of authors, the longer the horizontal axis of the ellipse extends. On the right side is the distribution cluster of cited journals, which represents the knowledge base cited by the current discipline. As shown in Figure 6, the cited journals are mainly distributed in themes #2 (MEDICINE, MEDICAL, CLINICAL) and #3 (NEUROLOGY, SPORTS, OPHTHALMOLOGY), while the cited journals are mainly concentrated in theme #5 (HEALTH, NURSING, MEDICINE), theme #7 (PSYCHOLOGY), theme #5 (MEDICAL), theme #5 (MEDICAL), and theme #7 (MEDICAL). 7 (PSYCHOLOGY, EDUCATION, SOCIAL) and theme #12 (ECONOMICS, ECONOMIC, POLITICAL). The colored curves connecting the left and right sides provide a complete picture of the citation paths, with the wider curves representing the major citation paths. NEUROLOGY, SPORTS, and OPHTHALMOLOGY are the most dominant citation groups, and are significantly influenced by the PSYCHOLOGY, EDUCATION, and SOCIAL disciplines (*z* = 5.356, *f* = 1,959).

### 3.7 Co-citation analysis of reference

**Figure 7:**
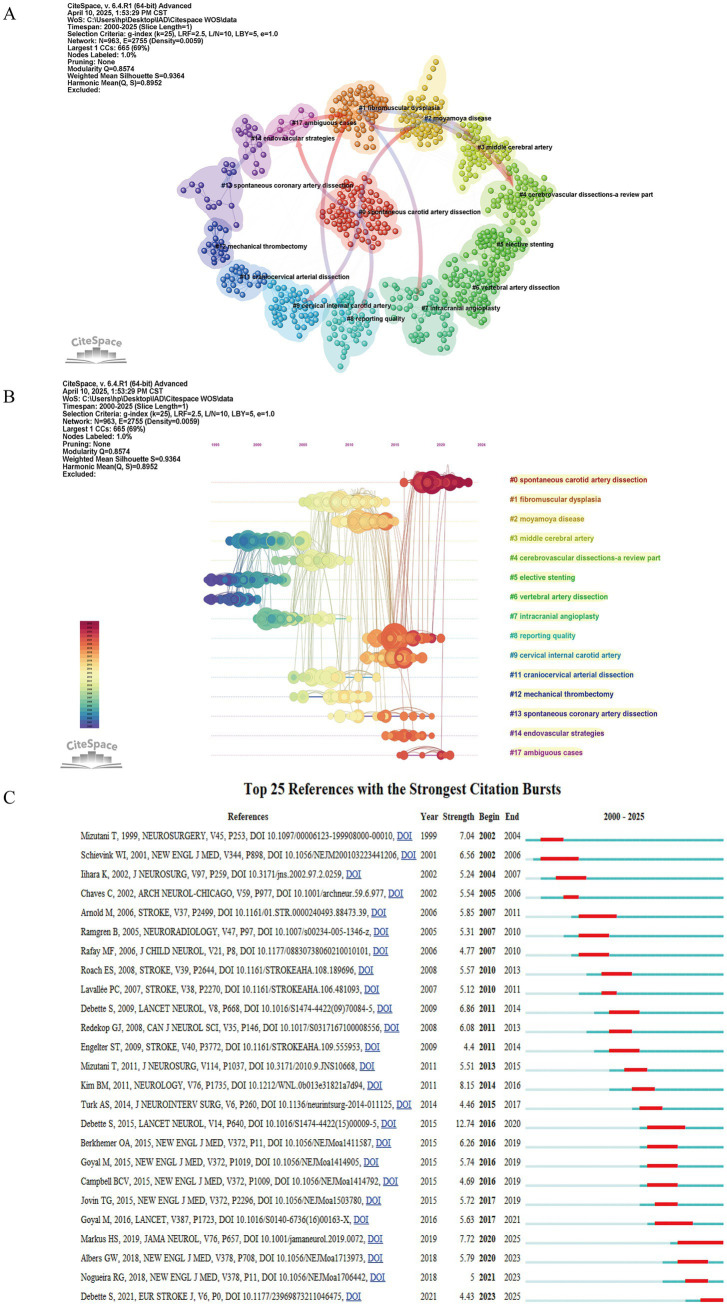
Cluster network analysis of references in the IAD research field from 2000 to 2024. **(A)** Visual analysis of the clustering network of co-cited literature. The top 15 largest citation clusters are shown. **(B)** Timeline view of co-cited references. Each horizontal line represents a cluster, with smaller labeled numbers and larger node sizes reflecting co-citation frequency, while links indicate co-citation relationships. The colors of the nodes and lines represent different citation years. **(C)** Top 25 references with the strongest citation burst.

Within this study, 1,130 articles were analyzed, collectively citing a total of 20,050 references. Table 6 provides a detailed account of the 10 articles that were cited most frequently. Many of these widely cited articles were featured in renowned journals specific to the field or in leading international publications, such as New England Journal of Medicine (JCR Q1, IF = 96.2), Lancet Neurology (JCR Q1, IF = 46.5), Stroke (JCR Q1, IF = 7.8) Neurosurgery (JCR Q1, IF = 3.9), Journal of Neurosurgery (JCR Q1, IF = 3.5). In March 2001, the team of Schievink M.D. published an article in the New England Journal of Medicine entitled “Spontaneous Dissection of the Carotid and Vertebral Arteries” is the most frequently cited article in this area, with 1,198 citations. Figure 7 shows a network visualization of literature co-citations using Citespace. Figure 7A shows the largest 15 reference clusters, with arrows marking the connections between clusters. The cluster modularity value (Q-value) of 0.8574 > 0.3 indicated that the cluster was structurally significant, and the cluster mean contour value (S-value) of 0.9364 > 0.7 indicated that the clusters were convincing. The smaller the numerical number on the cluster label, the larger the cluster, the greater the number of documents it contains, and the content of the label represents the subject matter of the clustered documents. Figure 7B shows a timeline plot of the evolution of the reference clustering, demonstrating how the citations of the references in each cluster change over time, with the size of the circular nodes representing the citation frequency of the literature. Figure 7C shows the top 25 documents with the greatest burst intensity, with the dark blue line segments representing the time frame in which the document was cited, and the red line segments representing the time when its citations exploded in growth. We can analyze the trend of citation volume to track the research hotspots in different time periods and predict the future research development of the field to some extent. References that exhibit high burst strength are likely to significantly influence future research. The top three references with the highest burst strength are Epidemiology, pathophysiology, diagnosis, and management of intracranial artery dissection (strength = 12.74, outbreak period 2016–2020) (3), Outcomes and prognostic factors of intracranial unruptured vertebrobasilar artery dissection (strength = 8.15, outbreak period 2014–2016) (8), Antiplatelet Therapy vs. Anticoagulation Therapy in Cervical Artery Dissection (strength = 7.72, outbreak period 2020–2025) (9). Between 2000 and 2025, we can roughly divide the period into 3 time periods based on the concentration of bursts and changes. From 2000 to 2006, the articles with increased citations were focused on exploring the causes and pathophysiological changes of spontaneous carotid and vertebral artery dissection, summarizing and analyzing the treatment and late follow-up of IAD and intracranial dissecting aneurysm (IDA) (2, 10, 11). From 2007 to 2017, researchers focused on the investigation of the pathogenesis of intracranial posterior circulation arterial dissection, and the analysis and summarization of clinical cases, as well as the clinical diagnosis and management strategies of stroke due to IAD in children (12–14). Cited literature from 2018 to 2025 focuses on endovascular treatment of ischemic stroke in the acute and hyperacute phases (15–17).

**Table 6:** Top 10 most cited references.

Rank	Title	DOI	Corresponding author	Citations	Journal	JCR	IF
1	Spontaneous Dissection of the Carotid and Vertebral Arteries	10.1056/NEJM200103223441206	Schievink W I	104	New England Journal of Medicine	Q1	96.2
2	Epidemiology, pathophysiology, diagnosis, and management of intracranial artery dissection	10.1016/S1474-4422(15)00009-5	Stéphanie Debette	58	Lancet Neurology	Q1	46.5
3	Recurrent subarachnoid hemorrhage from untreated ruptured vertebrobasilar dissecting aneurysms	10.1227/00006123-199505000-00003	Mizutani T	58	Neurosurgery	Q1	3.9
4	Clinical and Neuroradiological Features of Intracranial Vertebrobasilar Artery Dissection	10.1161/01.STR.30.5.1083	Takaaki Hosoya	54	Stroke	Q1	7.8
5	Dissecting intracranial aneurysms	PMID: 594878	Yonas H	49	Surgical Neurology	Q3	1.7
6	Dissecting aneurysms of the intracranial vertebral artery	10.3171/JNS.1990.72.2.0183	A Yamaura	48	Journal of Neurosurgery	Q1	3.5
7	Proposed classification of nonatherosclerotic cerebral fusiform and dissecting aneurysms	10.1097/00006123-199908000-00010	Mizutani T	46	Neurosurgery	Q1	3.9

### 3.8 Visual analysis of keywords

**Figure 8:**
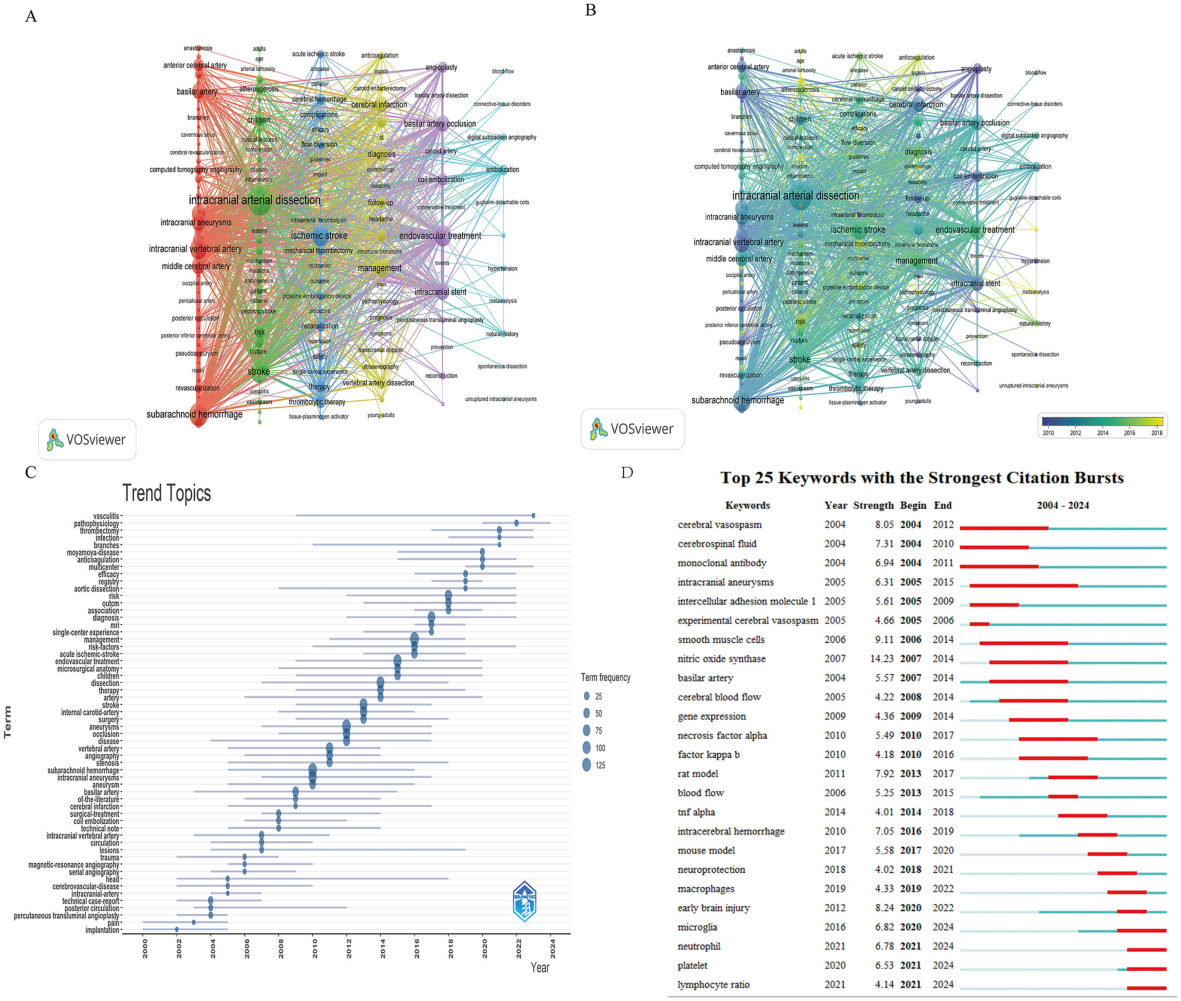
Visualization graph of keyword co-occurrence networks and evolutionary trends. **(A)** Co-occurrence network and clustering diagram of keywords. A total of 3,174 keywords, 154 with more than 5 occurrences were selected for visualization and analysis. **(B)** Stacked plots of keyword frequency versus time of occurrence. **(C)** Trends in research hotspots over time. **(D)** The top 25 keywords with the strongest bursts.

Cumulatively, 3,174 keywords were provided in 1,130 articles, and 154 keywords appeared more than 5 times. In Figure 8, the co-occurrence network and overlay visualization are depicted, with node sizes reflecting the frequency of word occurrences, and the lines linking the nodes illustrating the connections between the words. The top 10 keywords with the highest frequency of occurrence are listed in detail in Table 7, in which “intracranial arterial dissection” ranked first (*n* = 362), “stroke” second (*n* = 361) and “subarachnoid hemorrhage” was ranked third (*n* = 194), which were also the most closely related to the other keywords, and thus their importance can be seen. The clustering of the keywords is demonstrated in Figure 8A, where we can see that the 154 keywords with a frequency of greater than 5 occurrences are classified into 6 main clusters, which are shown in 6 different colors. The red cluster is the largest cluster, containing 44 keywords related to intracranial dissection aneurysms and subarachnoid hemorrhage. The green clustering is the second largest clustering and contains 38 keywords related to risk factors for IAD, diagnostic imaging, and pediatric stroke. The blue cluster included 25 keywords and was the third largest cluster with the topic of endovascular treatment of ischemic stroke. The yellow cluster included 23 keywords and the topic was antiplatelet drug therapy and prognosis of IAD. The purple and blue clusters were the two smallest clusters, including 13 and 11 keywords, respectively, and both clusters were related to endovascular stent and coil therapy for IAD. Figures 8B shows an overlay view of the keywords, overlaying the frequency of keywords with the average time of occurrence to clearly demonstrate the evolution of the keywords’ turnover over time in this research area with color shades. The darker the color of the node, the earlier it appears, and the lighter the color of the node, the later it appears. Keywords with purple nodes appeared in 2010 or earlier, and those with yellow nodes appeared after 2018. In the study of IAD, the research trend has gradually shifted from the symptoms, such as pain and subarachnoid hemorrhage, and endovascular treatment, to an in-depth investigation of the pathogenesis and pathophysiology of the disease, precise diagnosis by imaging and multicenter clinical trials. Figure 8C shows the display of changes in research trends according to keywords in the bibliometrics online analysis platform, which more intuitively shows the time of appearance of each keyword and the change in research heat over time. Figure 8D shows the 25 keywords with the highest citation bursts, and the top three keywords are “recanalization” (strength = 8.14), “risk” (strength = 7.68), and “basic artery” (strength = 7.17). According to the intensity and duration of the outbreak, we can see that the hotspots of attention of researchers in recent years are thrombectomy treatment, diagnosis and the exploration of risk factors.

**Table 7:** Top 10 most frequent keywords.

Rank	Keyword	Occurrences	Average citations	Cluster	Total link strength
1	Intracranial arterial dissection	362	20.8	2	1,566
2	Stroke	361	25.1	1	902
3	Subarachnoid hemorrhage	194	25.0	2	894
4	Intracranial vertebral artery	164	24.4	1	772
5	Endovascular treatment	163	20.9	5	884
6	Management	129	18.2	4	609
7	Intracranial aneurysms	109	26.7	1	503
8	Internal carotid artery	106	27.9	1	541
9	Intracranial stent	100	25.9	5	557
10	Middle cerebral artery	99	17.6	1	441

## 4 Discussion

### 4.1 Global trends

Between January 2000 and March 2025, 1,130 articles on IAD were screened in Web of Science and analyzed using CiteSpace 6.4. R1, VOSviewer 1.6.20, Microsoft Office Excel 2021, and the Bibliometrics Online Analysis Platform (see text footnote 1) were analyzed. Based on these scholarly article data, we assessed national, institutional, and author contributions, core articles, and research hotspots in this field of study.

In 1982, Harold P. Adams’ team reported the first case study of IAD, revealing the rupture characteristics of IDA by following two patients with pseudoaneurysms secondary to acute hemorrhage from arterial dissection (18). Since, then the research related to IAD has gradually heated up. Our analysis shows that although there was some upward fluctuation in the number of literatures of global IAD research, the results have remained at a high number in the last 15 years, suggesting that IAD is still one of the hotspots that researchers in the field of cerebrovascular disease are concerned about. Country analysis shows that the United States tops the list in terms of the number of publications and total citations, demonstrating its leading position and profound influence in the field, which is mainly due to the country’s continuous exploration of IAD and the continuous output of high-level research results. Such success can be credited to the substantial research funding, state-of-the-art facilities, and a robust academic framework that draws exceptional scholars from around the world to pursue their studies in the region. In terms of the institution’s academic outputs, the University of California System has the highest number of publications, followed by Assistance Publique Hôpitaux Paris (APHP), Capital Medical University, Universite Paris Cite, University of Toronto, and Mayo Clinic, all of which published more than 40 publications. In terms of the most influential journals, Stroke and Neurosurgery are at the top of the list with high citations and are important trendsetters in the field, as these journals publish highly regarded and cited research on IAD. In terms of scholarly impact, Biller J, Kirkham FJ, and Deveber G ranked in the top three with high citations, indicating that their scholarship is recognized by other researchers in the field. In terms of subject areas, CiteSpace’s journal overlay analysis revealed that current research on IAD exhibits significant interdisciplinary characteristics. Recent studies on IAD are primarily published in journals across these fields: MEDICINE, CLINICAL, SURGERY, NEUROLOGY or OPHTHALMOLOGY, as well as in other fields. On the contrary, the extensive use of HEALTH, NURSING, PSYCHOLOGY, EDUCATION, SOCIAL, ECONOMICS and other disciplines has improved the solid foundation of contemporary research. IAD research relies on multidisciplinary intersections and close global cooperation, with the theoretical backing of the basic sciences, the constant introduction of new ideas and methodologies and with the help of the translational capabilities of medical, the research results will be applied in practice, providing value for in-depth exploration of pathophysiological mechanisms and precise diagnosis and treatment of diseases, thus promoting the comprehensive development of the IAD field.

### 4.2 Research hotspots

#### 4.2.1 Continuous improvement in pathophysiology and risk factors research

Intracranial arteries have unique anatomical features, the lack of an outer elastic membrane, insufficient elastic fibers in the middle membrane, and the thinness of the outer membrane compared with extracranial vessels, making them hemodynamically susceptible. Significant hemodynamic stress, particularly turbulence associated with luminal irregularities, leads to structural deterioration of the arterial wall. The mechanism of acute IAD involves the invasion of circulating blood into the arterial wall through a portal formed by extensive disruption of the internal elastic lamina (IEL). This mechanism is applicable to all geometries and all clinical types of IAD. Notably, due to the lack of an extracranial vascular elastic layer as well as a thin tunica albuginea and a thin muscular layer, the intracranial site is predominantly characterized by subepithelial dissection (19–21). During this process blood enters the vessel wall, forming intramural hematomas and false lumen (11). However, connectivity with the intramural hematoma vessel lumen and evidence of intimal rupture were not found in autopsy studies of some patients with IAD, suggesting that the pathogenesis of IAD is not homogeneous. Trophoblastic vascular leakage between vessel walls may be one of the pathogenic mechanisms, but follow-up studies are still needed to refine the theory (22, 23). It is commonly believed that at the beginning of dissection formation, blood enters the subendothelial space to form an intermural hematoma. If an outlet exists at this time, a true lumen and a false lumen are formed, and the intermural hematoma in the false lumen may form a thrombus that travels distally through the outlet, leading to embolic stroke; if the intermural hematoma involves the beginning of the perforating vessel, perforator occlusion may occur. If no outlet exists, the intermural hematoma gradually accumulates and eventually leads to occlusion of the parent artery. If the hematoma breaks through the tunica, a pseudoaneurysm may form (24). In the study by George et al. arterial dissection in aneurysmal subarachnoid hemorrhage accounted for 8% of cases and the occurrence of subepithelial dissection may be an important factor contributing to subarachnoid hemorrhage (25). Due to the lack of epithelial tissue in the vessel wall to limit intermural hematomas, only one layer of thrombus prevents continued extravasation of blood after triggering a subarachnoid hemorrhage, leading to easy rupture of the dissecting aneurysms and a high re-rupture rate (24).

In addition, the increasing research on the pathophysiology of IAD has led to a clearer definition of the classification of IAD. Injury to the IEL is an important factor in IAD formation, and subepithelial dissection predisposes to pseudoaneurysm formation; thus, pseudoaneurysms can be categorized into four subtypes according to the degree of IEL pathology. Type 1: Classical dissection aneurysms with extensive disruption of the IEL and absence of thickening of the intima, including Ia, ruptured classical dissection aneurysms, and Ib, unruptured classical dissection aneurysms; Type 2: Segmental dilated IAD with IEL fragmentation and non-thrombosed intimal hyperplasia; Type 3: Dolichoectatic dissecting aneurysms characterized by IEL rupture and thrombosed lumen; Type 4: Saccular aneurysms unrelated to branching areas. These aneurysms occur in areas where the IEL is slightly disrupted without intimal thickening and are at risk of rupture. This classification system is closely related to the pathologic features and can provide the rationale for therapeutic options through the continuous and in-depth study of the pathologic mechanisms (2, 26).

The risk factors and mechanisms of IAD are still not fully understood, but migraine is an important predisposing factor for arterial dissection, probably because of the common genetic susceptibility (variants of PHACTR1 and its alleles) between migraine without aura and arterial dissection (27, 28). A study of young stroke patients showed that headache without aura was significantly associated with dissection (ratio [OR], 1.74; 95% CI, 1.30–2.33), but migraine with aura was not significantly associated with dissection. It has also been suggested by the inflammatory hypothesis that chronic (ictal and interictal) elevation of MMP levels in migraine patients may disrupt the arterial wall, making them more susceptible to IAD in conjunction with other factors (29). It is noteworthy that, in contrast to other types of cerebrovascular lesions, traditional atherosclerotic risk factors (including diabetes mellitus, hypercholesterolemia, and smoking) were less prevalent in patients with intracranial dissection than in extracranial vascular disease (31.8% vs. 64.0%, *p* < 0.05), suggesting a unique etiologic profile (30, 31). Dong et al. found that the prevalence of hypertension in patients with intracranial vertebral artery dissection (VAD) was significantly higher than that in the group with extracranial lesions (*p* = 0.005), and that hypertension may be involved in hemorrhagic transformation process by increasing the risk of intermural hematoma expansion, which increases the risk of bleeding in IAD (32). In addition, diseases affecting the arterial media are also strongly associated with arterial dissection, such as fibromuscular dysplasia (FMD) and cystic median necrosis (CMN) (33, 34). Abnormal vascular conformation is considered a key contributing factor. In a study of the relationship between cervicocerebral artery dissection and vascular tortuosity, it was mentioned that, the prevalence of tortuosity in dissected arteries ranged from approximately 22–65%, yet it was only 8–22% in non-dissected arteries (35). In tortuous cervical arteries, the degradation of elastin and the tunica media, increased wall stiffness, alterations in hemodynamics, and arterial wall inflammation may be associated with dissection. These findings provide a rationale for the development of prevention strategies based on hemodynamic assessment.

#### 4.2.2 Importance of diagnosis and identification

When the progression of IDA triggers ischemic or hemorrhagic events, it can be manifested by appropriate symptoms. However, most patients with IDA do not have specific symptoms, and radiological investigations can be used as key information for the diagnosis of IAD. Magnetic resonance angiography (MRA) and CT angiography (CTA) continue to be the most commonly used, and these investigations allow for the visualization of arterial vascular morphology. MRI combined with MRA is widely recommended as an initial screening tool for arterial dissection (36). Despite the progressive development of various imaging methods, digital subtraction angiography (DSA) remains the gold standard for the diagnosis of IAD.

Key imaging features of IAD, including the string sign (localized pathological stenosis of the vascular lumen), the string-and-pearl sign (proximal stenosis with distal dilatation), double lumen sign (showing both true and false lumens), intimal flap, pseudoaneurysm formation or cobblestone dilatation, intramural hematomas and contrast stagnation. These findings are critical for differential diagnosis and therapeutic decisions (37, 38). Emerging evidence emphasizes the utility of susceptibility-weighted imaging (SWI) in detecting intramural hematomas, particularly in vertebral artery dissection (VAD), with a sensitivity and specificity of over 90% for differentiating VAD from atherosclerotic lesions (39, 40).

Accurate diagnosis of true arterial dissection remains challenging due to low clinical detection rates. According to the European Stroke Organization (ESO) guidelines and a review in The Lancet Neurology (2015), the diagnosis of IAD should require at least one of the following 3 features: (i) stenosis or occlusion of intracranial arteries, with spindle or irregular aneurysm dilatation at a non-branching site; (ii) intramural hematoma, intimal flap, or double lumen; and (iii) pathological examination of the IAD (3, 17). The angiographic criteria for identifying dissections include the stagnation of contrast within the aneurysmal pouch, the presence of stenotic segments either proximal or distal to the ectasia and a more probable fusiform appearance (41).

The latest multicenter prospective cohort study published in Translational Stroke Research in January 2025 revealed a novel diagnostic model for symptomatic intracranial arterial dissection (sIAD), with a model of 7 serum biomarkers highly discriminatory for sIAD (area under the curve [AUC], 0.95). These 7 biomarkers, including 6 proteins and 1 cytokine, are involved in immune response and inflammation-related pathways with good concordance of expression levels between sIAD tissue and serum (42). This breakthrough provides a molecular typing basis for non-invasive screening of sIAD, and the novel circulating markers may hold promise for establishing a more cost-effective and accessible screening for IAD, which could help in clinical decision-making for sIAD.

Diagnostic criteria for acute headache or neck pain due to carotid or vertebral artery dissection based on the International Classification of Headache Disorders (ICHD-3), 3rd edition, suggests that sudden onset headache or neck pain is a core warning sign of intracranial artery dissection (43). Prospective cohort studies have shown that headache is the first symptom in 86% of patients with IAD and is often accompanied by localizing neurological signs: anterior circulation dissection tends to present with ipsilateral Horner syndrome or pulsatile tinnitus, whereas posterior circulation lesions are significantly associated with vertigo and nausea (44, 45). In the report of Simone et al. on the correlation between IAD and headache, they studied 419 patients with IAD, in which headache accounted for up to 70.4% of cases and was more common in the posterior circulation (68.2%) than in the anterior circulation (44).

#### 4.2.3 Continuous evolution of treatment technologies

Since the 1990s, endovascular intervention for vertebral artery dissection (VAD) has gradually entered the clinical field, with balloon test occlusion and percutaneous transluminal angioplasty as the main techniques (46). However, early stenting was difficult to adapt to clinical needs (47). With the gradual accumulation of cases (the number of reported cases worldwide grew rapidly from 2000 to 2016) and the iteration of technological updates, treatment strategies have gradually evolved toward individualization, including innovative solutions such as multilayer bare stenting, overlay stenting, and flow diverter devices (FDD) (48–51).

The Guideline for the management of extracranial and intracranial artery dissection, published by the ESO in 2021, for the first time systematically proposes a stratified treatment framework. Conservative treatment with antiplatelet agents in asymptomatic IAD patients. Among symptomatic IAD patients, revascularization is preferred for those with hemodynamic disorders. Patients presenting with acute ischemic stroke (AIS) are prioritized for intravenous thrombolysis (IVT) after excluding thrombolysis contraindications. In high-risk cases of combined subarachnoid hemorrhage (SAH), early intervention can significantly improve prognosis. Due to the lack of RCTs and the limited number of observational studies, the choice of the type of intervention for acute IAD-associated SAH should be derived from a multidisciplinary assessment. For those with IDA and isolated headaches, surgical intervention is considered only if imaging follow-up is abnormal or if compression symptoms are present (17).

In a single-center retrospective study, among 21 patients with hemorrhagic vertebrobasilar artery dissection, the 30-day mortality rate in the endovascular treatment combined with surgical treatment group (*n* = 14) was reduced by 57% (7.1% vs. 42.9%) compared with the conservative group (*n* = 7), and the rate of rebleeding was reduced from 43 to 0% (52). Notably, the introduction of FDD has further improved the safety of the treatment, as a recent meta-analysis of 329 patients with IAD showed that the proportion of pseudoaneurysms secondary to arterial dissection with a modified Rankin Scale (mRS) score of 0–2 at the last postoperative follow-up in patients with FDD reached 89.7%, with a rate of device-related complications of less than 4% (6).

Conservative treatment remains the main strategy for patients with IAD whose only symptom is isolated headache, as shown by Kobayashi et al., only 1 of 56 such patients (1.8%) required surgical intervention due to increased pseudoaneurysm volume after conservative treatment (mean follow-up 2.9 years), and no thromboembolic events occurred (53). However, the importance of imaging follow-up needs to be emphasized, and high-resolution MRI is recommended at least every 6 months to assess the stability of the dissection (54, 55).

Current decision-making regarding IVT for IAD-associated acute ischemic stroke remains clinically challenging, and ESO guidelines recommend avoidance of IVT for IAD-associated stroke based on theoretical bleeding risk (56). A recent study evaluating the risk of postoperative intracranial hemorrhage (ICH) in patients with AIS treated with IVT showed that the IAD group demonstrated a significantly higher risk of symptomatic intracranial hemorrhage (sICH) (OR 3.18; 95% CI 1.26–8.06) and worse functional prognosis than the non-IAD group (57). It is suggested that AIS patients with underlying IAD should avoid adapting IVT therapy to avoid high risk of sICH.

The use of FDD is becoming more common in current treatment strategies for IAD and has demonstrated unique advantages in the treatment of IDA. In a meta-analysis evaluating the safety and efficacy of FDD in the treatment of IDA in 329 patients, the final postoperative imaging follow-up showed a complete occlusion rate of 71.7% and an adequate occlusion rate of 88.3%, with a minimal need for re-treatment of 0.9% and a high technical success rate of 99.1% (6), which fully emphasizes the importance of FDD for the treatment of IDA. The study fully emphasizes the effectiveness and safety of FDD in the treatment of IAD, but more research evidence is still needed for its applicability to ruptured pseudoaneurysms. However, another study showed that FDD versus stents (with or without coiling) has not demonstrated significant differences in functional outcomes (mRS 0–2: 86.8% vs. 86%) and mortality (3.9% vs. 6%), but FDD had a lower recurrence rate (1.3% vs. 13.3%) (58). It’s worth noting that, most of the available evidence comes from observational studies and there is a lack of randomized clinical trials directly comparing different endovascular intervention techniques.

Treatment of IAD is also challenging because of its significant risk of recurrence. Recurrence is usually divided into three stages, early hemorrhagic recurrence, late non-hemorrhagic recurrence and chronic fusiform aneurysm transformation. A longitudinal study of symptomatic recurrence in 143 spontaneous IAD cases published in Stroke reported that at 8.2 years of follow-up, 47 patients experienced symptomatic recurrence, a recurrence rate as high as 33%, including hemorrhagic recurrence observed on day 4 after diagnosis (59). Understanding this triphasic recurrence and the corresponding histopathologic features can help determine treatment and follow-up strategies for patients with IAD.

## 5 Limitations

It is essential to recognize that our study has its limitations. Primarily, the data were exclusively drawn from the WoSCC database. While this database is extensively utilized for bibliometric analyses due to its broad and comprehensive scope, it may still omit certain literature. Second, the inclusion of only articles published between January 1, 2000 and March 15, 2025 may have resulted in the inclusion of articles published outside of this study’s timeframe of important research findings were overlooked. We only included literature in which the language was English for the study, resulting in the omission of research findings in non-English languages. In addition, the literature screening process relied on manual operations, and the subjective nature of the inclusion/exclusion criteria may have led to selection bias. Meanwhile, the continuously updating nature of the database makes it difficult for the current analysis results to fully reflect the dynamic whole picture of the research field. It is worth noting that bibliometric methods are inherently subject to time bias (earlier papers receive higher citations due to cumulative effects) and citation rate confounders (the phenomenon of self-citation), which may affect the objectivity of the analyzed findings.

## 6 Conclusion

This study thoroughly examines the current status and development trends in research on the pathophysiology and clinical treatment of IAD using bibliometric methods. The results of the study show that the study of the pathogenesis of IAD and its resulting endovascular treatment of IDA in terms of methodology and evaluation of effectiveness and safety has become an important research hotspot in recent years. With the continuous development and advancement of diagnostic and therapeutic imaging technology, and the gradual deepening of numerous clinical and basic studies, our knowledge of IAD has become more comprehensive, and there will be breakthroughs in future investigations. Continuous and comprehensive research in this field will lead to earlier and more accurate diagnosis, and therapeutic effects to improve patient prognosis.

## Data Availability

The original contributions presented in the study are included in the article/Supplementary material, further inquiries can be directed to the corresponding author.

## Footnotes

^1^ https://bibliometric.com/
